# Family Planning Counseling for Women Living with HIV in Low- and Middle-Income Countries: A Systematic Review of the Impact on Contraceptive Uptake, Intention to Use Contraception and Pregnancy Incidence, 2011 to 2022

**DOI:** 10.1007/s10461-024-04319-w

**Published:** 2024-04-25

**Authors:** Kevin R. O’Reilly, Ping Teresa Yeh, Caitlin E. Kennedy, Virginia A. Fonner, Michael D. Sweat

**Affiliations:** 1 Division of Global and Community Health, Department of Psychiatry, Medical University of South Carolina, Charleston, SC USA; 2 Department of International Health, Social and Behavioral Interventions Program, Johns Hopkins Bloomberg School of Public Health, Baltimore, MD USA; 3 FHI 360, Global Health and Population Research, Durham, NC 27701 USA; 4 Division of Global and Community Health, Department of Psychiatry, Medical University of South Carolina, 176 Croghan Spur Rd, Suite 104, Charleston, SC 29407 USA

**Keywords:** Family planning, Women living with HIV, Counseling, Low and middle income countries

## Abstract

Women’s ability to control their fertility and have the number of children they want when they want them is an internationally recognized human right. This right has been the driving force behind family planning programs in low- and middle-income countries for more than five decades. The HIV epidemic added greater urgency to those efforts once the risk of vertical transmission of the virus from mothers to their infants was recognized. In 2013, we published a systematic review of the evidence of effectiveness of family planning counseling for women living with HIV, emphasizing HIV related behaviors. In this updated review, we examined 23 studies, primarily from sub-Saharan Africa. The evidence we uncovered reflected efforts to integrate services provided to women. These showed that providing contraceptive services, including intensified counseling and support, in the HIV clinics where women living with HIV received their care increased the likelihood of subsequent use of modern contraception by as much as fourfold. These studies reflected a greater focus on women’s family planning decisions and behaviors and less focus on HIV-related behaviors. Among the possible causes of this noted difference we include the widespread coverage of antiretroviral treatment for HIV. This advance has apparently changed the rationale and the approach to integrating family planning and HIV services in ways that may not have been fully appreciated. The results, however, are beneficial: greater coverage of family planning for women who wish to control their fertility and a more equal partnership between family planning services and HIV services in pursuit of the mutual goal of providing integrated services to meet women’s needs.

## Introduction

Women’s right to control their fertility has been a recognized human right and an international public health priority for more than five decades. Family planning programs designed to provide women with information and contraceptive options have been heavily supported by donors throughout that period. When it became clear that mother-to-child transmission of HIV was an important source of new infections, the potential for enhanced access to reproductive health services and reducing mother to child HIV transmission led to promotion of family planning for women living with HIV [1–4].

To reify that integration, the World Health Organization and its partners in 2002 put forth a four-pronged strategy [5] which proposed interventions to prevent young women from acquiring HIV, to prevent women living with HIV from experiencing unintended pregnancies, to limit risk of mother-to-child transmission at the time of delivery, and to prevent acquisition of HIV by infants postpartum. That strategy guided HIV programs for more than a decade. Eventually, as it became clear that antiretroviral therapy (ART) could virtually eliminate risk of transmission of HIV from mother to child and also improved the health of people living with HIV, treatment for all became the dominant theme, decreasing the need for special emphasis on women of reproductive age to prevent vertical transmission of HIV [6]. The priority for family planning programs once again focused on helping women achieve their fertility goals.

In 2013, we published a systematic review of family planning for women living with HIV in low- and middle-income countries, covering studies from the years 1990 to 2011, which addressed family planning and HIV integration efforts principally designed to prevent vertical transmission of HIV [7]. We identified nine relevant articles, all from Africa, which showed that providing concerted information and support for family planning use, coupled with ready access to a wide range of contraceptive methods, was effective in increasing family planning use. However, effects on pregnancy overall were difficult to measure, and no studies assessed the effect of family planning on subsequent unintended pregnancy.

Integration of family planning into HIV services is now more commonly seen as an opportunity to provide women living with HIV with the ability to avoid unintended pregnancies. This development reflects increased treatment effectiveness, as well as growing emphasis on integrated health services more generally, to ensure a client-centered approach to care.

Here, we present an update of our systematic review with an expanded scope, recognizing that increased access and coverage of treatment for HIV has profoundly changed the context of interventions and programs. We assess the evidence for the impact of providing or promoting family planning services to women living with HIV on the outcomes of contraceptive uptake, intention to use contraception, and pregnancy incidence.

## Methods

### Objectives

This review is part of the Evidence Project, a series of systematic reviews of HIV behavioral interventions in low- and middle-income countries conducted jointly by the Medical University of South Carolina and the Johns Hopkins University Bloomberg School of Public Health. This review updates and expands our previous review on this topic published in 2013 [7]. We conducted this systematic review in accordance with the Preferred Reporting Items for Systematic review and Meta-Analyses (PRISMA) guidelines [8].

### Eligibility Criteria

Studies were included in the review if they met the following criteria: (1) family planning counseling (not just information or education) was provided to women living with HIV who knew their HIV status; (2) the intervention was conducted in a low-income, lower-middle income, or upper-middle income country (as defined by the World Bank [9]); (3) the intervention was evaluated using a study design that compared post-intervention outcomes using either a pre/post or multi-arm study design (including post-only exposure analysis); (4) the article was published in a peer-reviewed journal from May 2010 (the end date of the previous search on this topic) through June 21, 2022.

Studies presented data only from women living with HIV, or from both women living with HIV and without HIV. For the second category to be included in the review, the article must have presented pre/post or multi-arm data separately for women living with HIV. No language restrictions were used; English translations were conducted when necessary. If two articles presented data for the same project and target population, the article with the longest follow-up was retained for analysis.

### Search Strategy

We searched four electronic databases (PubMed, CINAHL, Sociological Abstracts and PsycINFO) following the search strategy presented in “Appendix”. Titles, abstracts, citation details, and descriptor terms were independently screened twice. Abstracts were used to screen for inclusion. Full-text articles were obtained which were then reviewed by two independent reviewers to select the final studies. Consensus was used to resolve any differences.

### Study Selection

A study staff member initially screened studies based on titles and abstracts and excluded non-relevant citations. Two senior study staff then screened the remaining citations using inclusion criteria. The results of both screenings were merged and compared, and consensus was established by discussing any discrepancies. Final study selection was based on a thorough reading of the full text articles.

### Data Extraction

Data were entered by two study staff members into a systematic coding form independently that included detailed questions on intervention, study design, methods, and outcomes. The two completed coding forms were compared, and discrepancies were resolved by consensus, with review and discussion with a senior team member as necessary.

### Risk of Bias Assessment

Risk of bias of included articles was assessed using an eight-point scale [10] which includes the following items: (1) prospective cohort; (2) control or comparison group; (3) pre/post intervention data; (4) random assignment of participants to the intervention; (5) random selection of subjects for assessment, or assessment of all subjects who participated in the intervention; (6) follow-up rate of 80% or more; (7) comparison groups equivalent on socio-demographic measures; and (8) comparison groups equivalent at baseline on outcome measures.

### Analysis

Due to differences in study design, type of intervention, and measured outcomes, we were unable to conduct meta-analysis. We therefore extracted key findings from each study and present them by intervention types offered or analyses conducted.

## Results

**Fig. 1:**
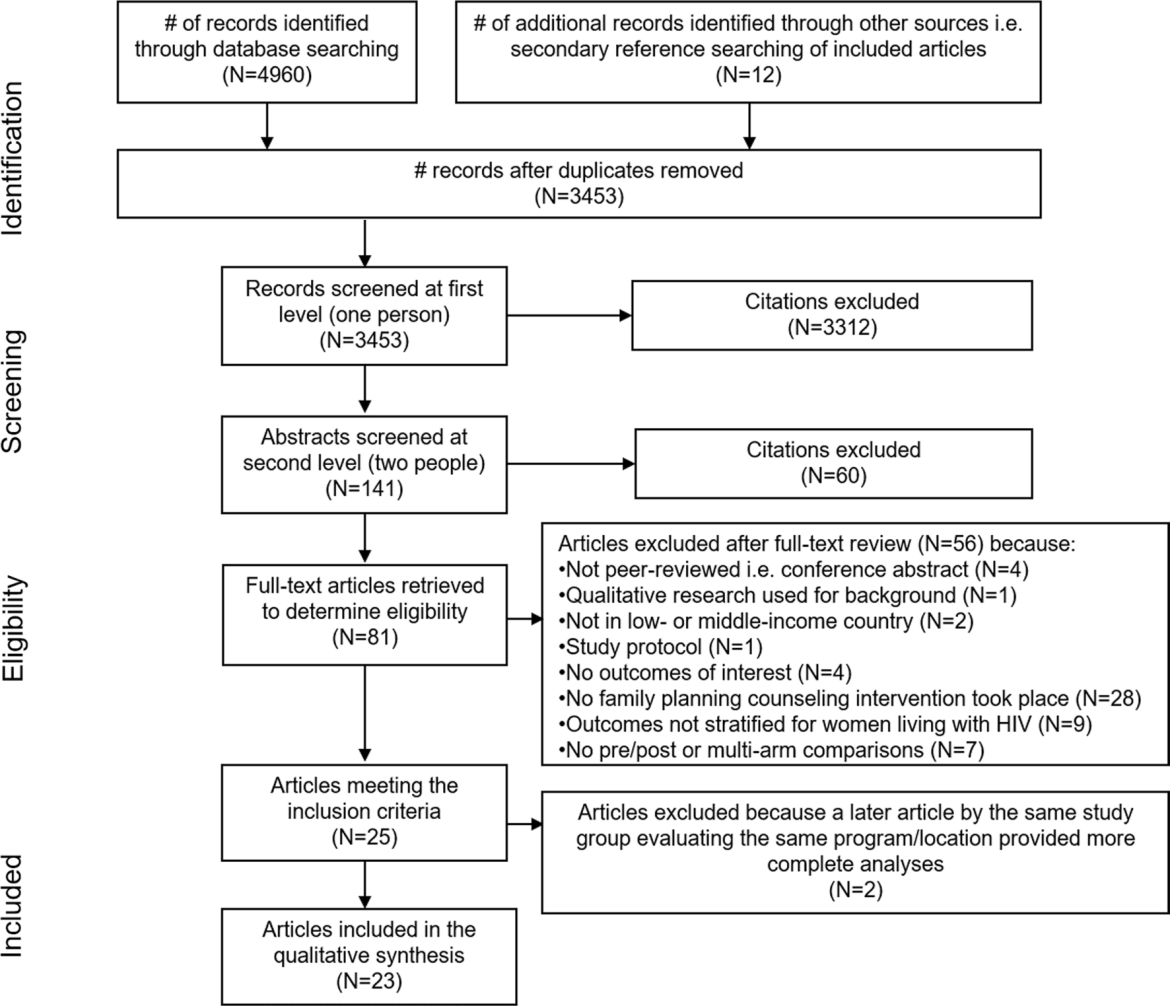
Flowchart of the systematic search and review process

Electronic database searching yielded 4960 potentially relevant citations (Fig. 1). An additional 12 possible articles were identified through other sources. Once duplicate records were removed, 3453 abstracts were reviewed by one screener and 3312 records were excluded as not meeting the inclusion criteria. The remaining 141 abstracts were then reviewed by two people who removed 60 abstracts. The full texts of the remaining 81 abstracts were reviewed by two people, of which 25 articles met the inclusion criteria [11–35]. However, two articles [13, 33] were excluded because they presented data that were duplicated and more fully presented in subsequent publications [12, 32], resulting in 23 articles included in the review.

All the included articles came from Asia or Africa. From Asia, there was one study each from Cambodia [30], India [19] and Thailand [22]. From Africa, there were six studies from Uganda [12, 13, 25, 26, 31, 34, 35], four from Kenya [15, 16, 20, 27], two from South Africa [18, 23] and one study each from Malawi [32], Botswana [17], Cameroon [21], Ethiopia [11], Nigeria [24], Swaziland [29], Tanzania [14] and Zimbabwe [28]. No comparative studies from other countries or regions were identified.

The assessment of risk of bias for included studies is presented in Table 1. Eight studies employed cross-sectional [11, 15, 26, 31, 35] or serial cross-sectional [14, 18, 25] study designs. Five studies used pre-/post designs [17, 21, 29, 30, 34]; two were retrospective cohort studies [32] and one used a time series design [22]. Four studies were randomized control trials [12, 23, 27] or cluster randomized trials [16], and three were nonrandomized control trials [19, 24, 28].

**Table 1:** Risk of bias assessment of articles included in systematic review

Study	Study design			Participant representativeness			Equivalence of comparison groups	
	Cohort	Control or comparison group	Pre/post intervention data	Random assignment of participants to the intervention	Random selection of participants for assessment	Follow-up rate of 80% or more	On sociodemographics	At baseline on outcome measures
Aradom et al. (2020) [11]	No	No	No	NA	Yes	NA	NA	NA
Atukunda et al. (2021) [12]	Yes	Yes	No	Yes	Yes	Yes	Yes	NA
Baumgartner et al. (2014) [13]	No	No	Yes	NA	No	NA	NA	NA
Dev et al. (2021) [14]	No	Yes	No	NA	No	NA	Yes	NA
Grossman et al. (2013) [15]	No	Yes	Yes	Yes	Yes	NA	Yes	Yes
Hawkins et al. (2021) [16]	No	No	Yes	NA	No	NA	NA	NA
Hoke et al. (2014) [17]	No	No	Yes	NA	No	NA	NA	NA
Joshi et al. (2016) [18]	Yes	Yes	Yes	No	No	Yes	Yes	NR
Kosgei et al. (2011) [19]	Yes	Yes	Yes	No	No	Yes	No	NR
Kuete et al. (2016) [20]	No	No	Yes	NA	No	NA	NA	NA
Landolt et al. (2017) [21]	Yes	No	Yes	NA	No	Yes	NA	NA
Mantell et al. (2017) [22]	Yes	Yes	Yes	Yes	No	No	Yes	Yes
McCarraher et al. (2011) [23]	Yes	Yes	Yes	No	No	Yes	No	No
Mudiope et al. (2017) [24]	No	No	Yes	NA	No	NA	NA	NA
Nabirye et al. (2020) [25]	No	Yes	No	No	No	NA	NR	NA
Onono et al. (2015) [26]	Yes	Yes	Yes	Yes	Yes	Yes	Yes	Yes
Sarnquist et al. (2014) [27]	Yes	Yes	Yes	No	No	Yes	Yes	Yes
Siveregi et al. (2015) [28]	Yes	No	Yes	NA	No	NR	NA	NA
Thyda et al. (2015) [29]	No	No	Yes	NA	No	NA	NA	NA
Tusubira et al. (2020) [30]	No	Yes	No	No	Yes	NA	NR	NR
Tweya et al. (2018) [31]	Yes	No	Yes	NA	No	NR	NA	NA
Vu et al. (2017) [32]	Yes	No	Yes	NA	No	No	NA	NA
Wanyenze et al. (2015) [33]	No	Yes	No	No	No	NA	NR	NR

The included studies fell into two general types. The first type implemented and tested a specific intervention, while the second type focused on evaluating policy/programmatic studies, including national policy, clinic-level strategies or integration more generally. The thirteen studies in the first group are presented in Table 2 which describes the nature of the intervention provided as well as its effect. The eight descriptive or evaluative studies in the second group are presented in Table 3. Two additional studies which focused on men are presented separately in the text.

**Table 2:** Studies presenting outcome data on current contraception use, unmet need for family planning (FP), intentions to use family planning and incident pregnancies following intervention

Study/location/years of study	Study design/outcomes measured	Comparison	Intervention	Effect
Atukunda (2021) [12] Uganda October 2016–June 2018	Randomized control trial Continuous use of contraception, pregnancy incidence at 12 months postpartum	Structured and sustained family planning support (160 women at baseline, 158 at 6- and 12-month follow-up) compared to 160 women in standard care (159 at 6- and 12-month follow-up	Structured, sustained FP support: following delivery, women in intervention group were counselled and given FP voucher. Structured immediate postpartum counselling offered in private room by nurse, lasting up to 40 minutes. A well-trained nurse was available at post-natal clinic to identify women with vouchers to access the relevant service provider within one hour of arrival. Minimized stock outs. Daily scheduled SMS reminders for women who chose OCs for first 4 months, then weekly reminders the next 2 months. Sent monthly if injectable was chosen	Confirmed continuous use FP: Standard care 69.2%, enhanced care 79.8% (OR 1.75, 95% CI 1.24–2.95) Pregnancy in first year postpartum: Standard care 8.8%, enhanced care 1.9% (OR 0.20, 95% CI 0.05–0.62)
Baumgartner (2014) [13] Tanzania September 2009–February 2010	Serial cross-sectional study Contraceptive method use, unmet need, dual method use	323 women pre intervention compared to 299 post intervention	Facilitated referral, with seven service delivery steps: (1) screen all female clients for fertility intentions and current use, (2) counsel on options, (3) refer for FP services, (4) record referral, (5) accompany to FP clinic, (6) access FP services, and (7) monitor and follow-up	Proportion of sexually active women using contraceptive method increased 12% (p = 0.013) Unmet need decreased by 4% Dual method use increased 16% (p = 0.014)
Grossman (2013) [15] Kenya December 2009–September 2011	Cluster randomized trial Use of effective contraceptive methods, Pregnancy rates	Twelve clinics integrated FP services into HIV clinic, six were controls referring clients to FP clinics at the same facility	FP integrated into HIV clinics, compared to control clinics which referred clients to separate FP clinics at the same facility	Women at integrated sites more likely to use more effective contraceptive methods (increase from 16.7 to 36.6% at integrated sites, 21.2% to 29.8% at control sites; OR 1.81 CI 1.24–2.63). Condom use decreased at integrated sites compared to controls (OR 0.64, CI 0.35–1.19 NS). No difference in incident pregnancies
Hawkins (2020) [16] Botswana October 2017–August 2018	Prospective, hybrid type 2 clinical intervention and implementation study Contraceptive use and preference for A. *very effective* (intrauterine device (IUD), implants, tubal ligation) B. *effective* (3-monthly injectable, oral contraception) C. *less effective* (male and female condom)	141 women pre-intervention to 107 women post (same clinic visit)	FP services integrated into HIV clinic: Brief training on contraceptive counseling plus option of immediate referral to on-site provider	Significant increase (p < 0.001) in the proportion of women interested in more effective contraception. No adjustment factors
Hoke (2014) [17] South Africa 2009–2010	Serial cross-sectional study Consider using IUD or tubal ligation in future	Two separate groups of women, 538 prior to and 539 after intervention implementation	Reinforcement of counselling on postpartum FP; providers trained on IUD and female sterilization; trained on IUD insertion; improved supply management; referral for female sterilization; on-the-job coaching and mentoring provided; information/education/communication materials and job aids	IUD and sterilization not affected by the intervention
Joshi (2016) [18] India July 2011–December 2013	Cohort study with pre- post- comparison Changes in awareness and knowledge, accessing FP services, acceptance of dual contraceptive methods, fertility desires and reported pregnancies	Experimental group—study site where a set of interventions were implemented to improve use of dual method and link HIV and FP services compared to a study site with on intervention, only routine standard care; 150 women in each arm	Linked HIV counselling and testing services with family planning services in the intervention clinic; routine standard of care in comparison clinic. Intervention package included one day training of service providers of counselling and testing, of PMTCT services and FP services, development and display of posters on dual methods, providing counselling on dual methods, operationalising a referral mechanism to FP clinics within the hospital set-up, among others	Women in the enhanced intervention clinics received counseling and testing for HIV, and promotion of dual methods, follow-up, and referral to family planning services within the hospital and other management support activities. Women attending the integrated clinic compared to controls demonstrated increased knowledge on injectable contraceptives (96.5 vs 53%), female condoms (44 vs 18%), and emergency contraceptive pills (81.6 vs 30%). Sixty percent of integrated clinic attendees reached family planning services after referral compared to 8% in the control arm
Kosgei (2011) [19] Kenya October 2005–February 2009	Retrospective cohort New FP use including condoms, new FP excluding condoms, new condom use, new pregnancies	Records of 1453 women attending enhanced care, 2578 attending regular care All data derived from existing clinical data	Nurses experienced in FP relocated to HIV care team; Reproductive Health Room integrated into patient flow, with “some degree of independence maintained for both FP and HIV care”; routine offer of same-day “one-stop shopping” appointments w/ central check-in/out; use of same patient charts; consistent messaging. All FP methods except surgical sterilization offered through the HIV clinic	New FP including condoms increased significantly in integrated arm (incidence 12.9%, p < 0.001) New FP use excluding condoms decreased in integrated arm (incidence 3.8%, p < 0.001) New condom use increased significantly in integrated arm (incidence 16.7%, p < 0.001) No reduction in incidence of pregnancy
Kuete (2016) [20] Cameroon February 2014–December 2014	Before-after study Intention to use FP	94 women interviewed first trimester and two weeks prior to delivery	Counseling about PMTCT and postpartum contraception provided two weeks prior to delivery. Other FP services not integrated	Intention to use FP increased from approximately 27% to nearly 80%
Landolt (2017) [21] Thailand June 2013–August 2015	Time series study Use of effective contraception, dual method use	77 female adolescents living with HIV screened at baseline, 70 followed for 12 weekly follow-ups (to week 48)	Guided reproductive health education through video, brochures, and individual counselling; offered free effective contraception in addition to dual protection	Screening 21% dual method Baseline visit after educational activities 55% dual method (14% with LARC) (p < 0.001) Week 24: 71% dual method (20% with LARC) (p = 0.0027) Week 48: 74% dual method (31% with LARC) (n.s.)
Mantell (2018) [22] South Africa No date reported	Group randomized trial Adherence to safer sex guidelines, or safer conception guidelines for women seeking pregnancy	Three session provider-delivered enhanced intervention with on-site FP services compared to existing standard of care	Three-session provider-delivered enhanced intervention with on-site contraceptive services to increase adherence to safer sex guidelines for women who did not wish to become pregnant or to safer conception guidelines for women seeking pregnancy	At least 10% greater adherence in the intervention arm than in the standard of care arm
McCarraher (2011) [23] Nigeria 2008–2009	Non-randomized trial Modern contraception use	335 women at baseline, compared to 274 women at 12–14 months follow-up	Enhanced integration: training of FP providers w/ updated info on FP, counseling and meeting FP needs of HIV-pos clients; enhanced facility level support including observing FP counseling sessions; reviewing M&E forms and facilitating meetings between HIV and FP providers and community volunteers to discuss and resolve issues; community mobilization; use of volunteers to escort ART clients to FP services (no evidence of onsite provision of FP at HIV clinics	Reported consistent condom use increased significantly from 1 to 12% in basic group and from 15 to 23 percent in the enhanced group Adjusted difference in change between enhanced intervention and comparison was -0.6% (NS) Referral mechanism appeared to fail: only 26% in the basic group and 33% in the enhanced group at follow up reported being referred to FP services for noncondom methods
Mudiope (2017) [24] Uganda 2012	Serial cross-sectional study Uptake of FP, unmet need for FP	Tracks average weekly attendance over three periods: pre-intervention (3 months), intervention (6 months), post-intervention (3 months)	FP counseling and education delivered by peers (mothers living with HIV); referral to adjacent FP clinic	Improved uptake of FP at 6 months (31.3%, p < 0.001 int vs. control) then fell (by 10.8% p = 0.005) in post intervention assessment Improved identification of mothers living with HIV in need of FP, improved referrals for FP
Sarnquist (2014) [27] Zimbabwe May 2011–August 2011	Non-randomized trial Uptake of LARC, Women’s control over condom use, Sexual negotiation power and ability to advocate for FP	Intervention group of 65 women compared to standard of care group of 33 at baseline, with overall retention of 96% over the 6-week postnatal and 3-month well-child visits	Three 90-minute group sessions at one clinic, focusing on sexual negotiation skills and empowerment, info about HIV, PMTCT and FP, and communication skills related to sex and FP. discussions, behavior modeling songs/drama, role playing were used. Providers (nurses) were educated in providing modern FP options, including insertion and removal of LARC	No difference in uptake of contraceptives between groups at baseline and 6 weeks 3 months postpartum: increased knowledge about IUD in intervention group (p = 0.002), more power (p = 0.01), more control over condom use (p = 0.002) and increased likelihood of disclosure of HIV status (both directions) p = 0.04 Combining intervention and standard-of-care groups (no difference between them) and comparing pre-most recent pregnancy and 3 months postpartum: significant increase in LARC (p < 0.001)

**Table 3:** Studies presenting evaluative data on policy or programs to address current contraception use, unmet need for family planning (FP), intentions to use family planning and incident pregnancies without providing specific interventions

Study/location/years of study	Study design/outcomes measured	Comparison	Description/evaluation	Effect
Aradom (2020) [11] Ethiopia April 2017–June 2017	Cross sectional study Use of modern contraception	Women who reported receiving FP counseling in HIV clinic, compared to those who reported not receiving counseling	Assessed frequency and predictors of use of modern contraception among women attending chronic HIV care and treatment clinics	Women who received counseling about modern FP from their ART provider were 4.53 times more likely to use modern FP than their counterparts (adjusted OR (95% CI): 4.53 (1.7–12.06))
Dev (2021) [14] Kenya June 2016–September 2016	Cross sectional study Contraceptive use in last month, intention to use contraception	Contraceptive counseling with HIV care provider at HIV Care and Treatment Centers vs. no FP counseling in the last year	Assessed clients’ recall of having received counseling. No additional intervention provided	Contraceptive use in the last month: Intervention: 2626/4697 (93%) vs. Control: 1453/4697 (78%) Adjusted OR (95% CI): 1.74 (1.41–2.15); p < 0.001 Intention to use contraception: Intervention: 114/577 (58%) vs. Control: 130/577 (34%) OR (95% CI): 1.85 (1.40–2.44); p < 0.001
Nabirye (2020) [25] Uganda August 2016–November 2016	Data drawn from quantitative national cross-sectional survey Missed opportunities to receive FP counseling; effect of receiving counseling on current use of modern FP	Contraceptive use among women in HIV care not currently pregnant and not intending pregnancy who received family planning counseling compared to those who did not receive family planning counseling	Data drawn from national cross-sectional survey of 5198 women receiving care at 245 HIV clinics, assessing the proportion of HIV positive women who missed FP counselling and whether receipt of counselling increased use for women who did not desire more children	One quarter of the women surveyed reported not receiving FP counselling. Those receiving FP counselling were significantly more likely to report modern contraception use (adjusted PR (95% CI): 1.21 (1.10–1.33))
Siveregi (2015) [28] Swaziland February 2014–May 2014	Pre-post study Current use of contraception	Before and after a 20-minute face-to-face counseling session, 711 women	Assessed the effect of counseling for neg and pos women on use and choice of FP method, unintended pregnancy rates, future fertility and reasons for contraceptive choices	Current LTPM use by those with previous counseling prior to study visit vs none: Log regression coefficient: 0.91 (p = 0.002) Those with previous counseling 2.5 times more likely to use LPTM than those without prior counseling In logistic regression, only level of education and prior LTPM use were associated with LTPM preference after counseling
Thyda (2015) [29] Cambodia July 2011–July 2012	Pre-post design Current use of non-condom contraceptive method, Use of dual protection	250 women involved in sex work and entertainment pre-integration compared to 249 women post-integration	Measured changes in knowledge and self-reported uptake of contraception before and after implementation of contraception/FP services at a peer-managed HIV services clinic	Pre-integration 84.6% reported using condoms exclusively. No significant increase in proportion using non-condom contraception after integration. No significant change in use of dual protection
Tusubira (2020) [30] Uganda April 2016–May 2016	Cross sectional study Current use of a modern contraceptive method	369 women postpartum FP counseling during antenatal care vs none	Cross sectional survey of women living with HIV who had delivered in the previous 2 to 18 months	Current use of family planning(?) among all women: Intervention vs. control: adjusted PR (95% CI): 1.53 (1.07–2.18) Among married women or in consensual union: Intervention vs. control: adjusted PR (95% CI): 1.41 (0.99–2.02) 33% had unmet need for modern methods Unmet need for spacing (24%) higher than for limiting births (9%).
Tweya (2018) [31] Malawi January 2012–December 2016	Retrospective cohort study Contraceptive use, Pregnancy rate	Longitudinal comparison on women using electronic medical records	Cohort analysis of the effect of integration of FP services into HIV care clinic	Among 10,472 women (15,700 person-years of observation), contraceptive use increased from 28% in 2012 to 62% in 2016 (p < 0.001). Rates of pregnancies decreased from 6.8 per 100 person-years in 2012 to 1.3 per 100 person-years in 2016 (p < 0.001)
Wanyenze (2015) [33] Uganda February 2011–June 2011	Cross sectional study Current use of FP, Current use of any modern FP, Current use of effective FP, Overall unmet need	408 women attending clinic with minimal FP information vs 389 women attending clinic with FP integrated into HIV care	Compared one clinic with fully integrated FP services and methods provided on site to a Catholic hospital clinic with information provided but no services and no referral	Overall, 58.2% reported using effective modern method of FP, lower in basic clinic (50%) than in integrated (57.9%); p = 0.04 Unmet need for limiting childbirth in basic clinic (41%) and integrated (31.7%) not significantly different Unmet need for child spacing in basic clinic (51.6%) significantly higher than in integrated (30.1%) (p = 0.008)

*ART* antiretroviral therapy, *CI* confidence interval, *FP* family planning, *LTPM* long term and permanent methods, *OR* odds ratio, *PR* prevalence ratio

Taken as a group, the included studies presented in both tables used a wide variety of outcome measures to assess outcomes, both at the individual level and at the macro level. Sixteen of the included studies measured some aspect of current contraception use [11–16, 19, 21, 23, 24, 27–31, 33], three measured unmet need for family planning [13, 24, 33], three captured some aspect of intentions to use family planning [14, 17, 20], and four measured incident pregnancies following an intervention or integration [15, 18, 19, 31]. The wordings and specifics of the measures used and the results obtained are included in Tables 2 and 3.

The interventions provided in these studies included in Table 2 can be grouped in three large categories. Nearly all of these studies provided intensified support for family planning use, including individual counseling. Only three studies did not mention this as an element of the intervention offered [13, 15, 19]. Five studies focused efforts on integration of family planning and HIV services, including on-site provision of family planning methods either in the HIV clinic or at a family planning clinic in the same complex [15, 16, 18, 19, 23]. One study tested a facilitated referral model [13].

Increases in use of modern contraception following an intervention were reported in four studies, of which three provided intensified support for family planning [12, 21, 24] and one provided facilitated referral [13] (Table 2). In Uganda, Atakunda et al. [12] reported a significant increase in continuous use of family planning in the enhanced care group receiving structured sustained family planning support compared to standard care (OR 1.75, 95% CI 1.24–2.95). Landolt et al. [21] found a significant increase in dual method use among sexually active adolescents in Thailand, as well as increasing use of long-acting reversable contraception up to 48 weeks after initial visit. Mudiope et al. [24] reported a significant 31.3% increase in uptake of family planning at 6-month follow-up among women receiving the intervention compared to controls (p < 0.001) but a subsequent decrease in the post-intervention period. Baumgartner [13], testing a facilitated referral model, reported a significant 12% increase (p = 0.013) in the proportion of sexually active women using a contraceptive method in Tanzania. In a study in India, Joshi et al. [18] found that sixty percent of integrated clinic attendees reached family planning services after referral compared to only 8% in the control arm.

One intervention study differentiated subsequent use of family planning by women’s fertility intentions. Asking women living with HIV if they desired to be pregnant in the following 12 months, Atukunda et al. [12] found low levels of desire for future pregnancies in the intervention group compared to the control group (OR 0.23, 95% CI 0.08–0.64, *p* = 0.002). Other studies addressed the issue less directly, either not reporting percentages of women who desired pregnancy [13] or identifying women “in need of family planning” without specifying if that was subjectively or objectively defined [21, 24]. Baumgartner [13] assessed the effectiveness of the facilitated referral intervention in reducing unmet need for family planning and found a small statistically insignificant 4% decrease.

Three of the intervention studies assessed subsequent pregnancy. In Uganda, Atukunda et al. found a significant decrease in pregnancy in the first year postpartum after intervention [1.9% in enhanced care versus 8.8% in standard care (OR 0.20, 95% CI 0.05–0.62)] [12]. The two other studies did not detect a difference in subsequent pregnancies following intervention [15, 19].

The studies that primarily described or evaluated aspects of integration (Table 3) reported on much the same outcomes as the studies that implemented and tested specific interventions. In Ethiopia, Aradom et al. [11] reported an adjusted odds ratio of 4.53 (95% CI 1.7–12.06) for use of modern contraception among women receiving counseling about family planning from their ART provider compared to those not receiving counseling. In a retrospective cohort analysis in Malawi, Tweya et al. [31] examined the effect of the integration of family planning services into a large ART clinic in 2011. Using electronic medical records, the authors found that contraceptive use increased from 28% in 2012 to 62% in 2016 (p < 0.001). Pregnancy rates decreased over the period, from 6.8 per 100 person-years in 2012 to 1.3 per 100 person-years in 2016 (p < 0.001). In a cross-sectional study comparing use of modern family planning methods by women attending a clinic with minimal family planning information compared to women attending a clinic with family planning integrated into HIV care in Uganda, Wanyenze et al. [33] found a small but significant difference: 57.9% of women in the integrated clinic used a modern method compared to 50% of women in the clinic with only basic information (p = 0.04). In Swaziland, Siveregi et al. [28] found that most women were using a modern contraceptive method, with use among women living with HIV (84%) higher than among women without HIV (72.3%). After counseling, use of long term or permanent methods increased from 15.3 to 42.4% among women living with HIV.

Only two studies included men. In a study nested within a cluster randomized trial in western Kenya, Onono et al. [26] assessed the impact of integrating FP and HIV services on women’s and men’s knowledge of and men’s attitudes toward family planning. In general, familiarity with family planning methods was relatively high among women at baseline and increased by endline. No difference was detected between the fully integrated versus nonintegrated services. No significant changes in knowledge scores for men were found over time nor by integration status of the clinic attended. In a study in Uganda, Vu et al. [32] measured the effect of peer support groups and peer-delivered interventions for youth living with HIV, both male and female, on a variety of key behaviors. After adjusting for gender, age, education, marital status, and relevant covariates in a multiple regression analysis, Vu et al. found significant increases in self-efficacy for condom and contraceptive use (adjusted odds ratio [AOR] 1.82, 95% CI 1.30–2.55), knowledge about HIV (AOR 1.83, 95% CI 1.29–2.61), condom use at last sex (AOR 1.72, 95% CI 1.18–2.51), disclosure of HIV serostatus to a sex partner (AOR 1.61, 95% CI 1.01–2.55) and use of modern family planning methods (AOR 1.7, 95% CI 1.1–2.7), among others.

## Discussion

Most of the studies reviewed reported significant results that were moderately effective in increasing family planning use among women living with HIV in low- and middle-income countries. Not all interventions evaluated were effective, however. Given the range of contexts for these studies, the variety of interventions and the complex challenge they address, that finding is not surprising.

Overall, across a variety of research designs, integration of services was found to be effective either in increasing some measures of current use of modern contraception [11, 14–16, 19, 28, 30, 31] or in increasing intention to use modern contraception in the future [14, 20]. Providing contraceptive methods on site in integrated clinics was effective in increasing use of modern contraception in many studies, resulting in increases ranging from the modest to more than doubling the rate of the comparator [12, 15, 19, 27]. This was not the case in all studies. In South Africa, for example, an intervention failed to increase acceptance of an intrauterine device (IUD) provided on-site or of tubal ligation which required referral [17], and in Cambodia, the target population of sex workers remained steadfast in their use of condoms and did not adopt dual protection by accepting modern contraception [29]. Referral for family planning services was also effective in increasing contraception use in some but not all studies. In Tanzania, a facilitated referral model yielded a 12% increase in use of modern contraception [13], and a small study in Uganda found the referral model resulted in the majority of referred women accepting at least one effective family planning service offered [24]. In another study in Nigeria, referral efforts appeared to be largely ineffective [23]. In general, the immediate availability of family planning services on-site has the logical appeal of being one less barrier for women whose fertility intentions were clear to them. The empirical evidence reviewed here by and large supports that logic.

The promotion of family planning for women living with HIV has garnered considerable interest in recent years. Two recent systematic reviews have been published, one addressing the factors that facilitated or constrained the integration of family planning into HIV services [36], the other examining whether integration of FP and HIV services increases the uptake of contraception among all women regardless of HIV status [37]. Our current review evaluating the impact of such programs adds to that growing literature.

Originally intended to be an update of our previous systematic review [7], this review differs in important ways. The priority given to PMTCT, considered essential a decade ago and prominent in our previous review, has decreased as effective HIV treatment has become widely available in low- and middle-income countries. Our previous review focused on the impact of family planning counseling and services on key HIV risk behaviors, including the prevention of mother to child transmission. The studies in this review primarily focused on family planning and women’s contraceptive use to achieve their desired fertility goals. Specific HIV-related outcomes were less in evidence, especially in the later studies. The numerous family planning outcomes addressed came with increased varieties of measurement employed.

The range of interventions evaluated also increased beyond what we encountered previously. An emphasis on integration of FP services in HIV treatment settings was clear, either through direct offer of services or through referral. Some studies provided enhanced interventions as well, to motivate family planning acceptance and use among women living with HIV.

Our earlier review found only nine articles meeting similar inclusion criteria over a period of 21 years [7]. This updated review found 23 articles over 12 years, with more diversity in the countries where the research took place. Though involving men in reproductive health decisions and services has long been considered an important goal and remains so [36, 37], only two of these studies included data from men. We clearly may have missed studies that focused exclusively on men, as that was not an element of our inclusion criteria. However, the fact that so few of the papers involving women included both genders in recognition of the important role that men may play in contributing to, facilitating or impeding women’s decision to use family planning is noteworthy. This paucity of data on the role that intervening with men might have on enhancing uptake of family planning by women is a weakness of the extant literature.

The challenge faced by many of these studies was facilitating future intentions and decision- making by women faced with complex social, cultural, and health issues. Each of these studies endeavored to detect the effectiveness of interventions that tried, through various means, to provide information, motivation, counseling, support, and access to the means to prevent unintended pregnancies. Their success in increasing contraceptive use may be considered modest given the level of effort and attention provided to the women who participated. The complex intersection of fertility desires and intentions, access to needed support and methods, as well as perceived limits on women’s reproductive rights and on their agency once fertility intentions have been formed, all conspire to make the challenge that much greater.

Decisions about fertility are complicated and often not solely under the control of an individual woman. In the presence of HIV infection, which heightens the importance of well-informed decisions about future fertility, the complication increases, influenced by the health status of the woman and her partner, previous parity, her openness about her HIV status, expectations of longevity, and concerns or experiences of HIV-related stigma, to name but a few. That these interventions have helped women as much as they appear to have done is a notable accomplishment. Recognizing the many factors that impinge on women’s ability to fully exercise their reproductive rights is the focus of reproductive health justice [38]. The importance of an enabling environment that allows women to fully exercise their right to decide when, how, how often, with whom they have sex and with which outcomes is increasingly being recognized. The absence of such an enabling environment will limit women’s ability to act on even the best counseling and support they may receive through efforts like the ones included in this review. The limited role of men in these interventions, addressed only in two of these studies, underscores the important need to address their continued influence, either facilitating or impeding, in the development of such an enabling environment.

## Conclusions

The development of integrated health services has become a goal within global public health. For women, integrating family planning and HIV services has garnered considerable attention. Originally driven by the need to prevent mother-to-child transmission of HIV, a key goal of HIV programs, that integration is now driven largely by the desire to help women achieve their fertility goals and protect their reproductive health. As such, family planning and HIV integrated services now are increasingly regarded as nearly equal partners, a laudable advance from the previous era when FP was largely a helper service to achieve an important HIV goal. In this review, we found that integrated FP and HIV services can help women living with HIV grapple with their complicated decisions about fertility and take the steps necessary to act on those.

## Data Availability

Available upon request to the corresponding author.

## Appendix

Search terms, by electronic database

**Table:**

Pubmed	("family planning" OR "birth control" OR contraceptive OR contraception OR fertility OR "reproductive counselling" OR "reproductive counseling" OR "reproductive planning" OR "birth spacing") AND (HIV-infected OR HIV-positive OR "women living with HIV" OR "people living with HIV" OR "HIV seropositive" OR "infected with HIV" OR "living with AIDS" OR "HIV seropositivity")
Embase	('family planning' OR 'birth control' OR contraceptive OR contraception OR fertility OR 'reproductive counselling' OR 'reproductive counseling' OR 'reproductive planning' OR 'birth spacing') AND (HIV-infected OR HIV-positive OR 'women living with HIV' OR 'people living with HIV' OR 'HIV seropositive' OR 'infected with HIV' OR 'living with AIDS' OR 'HIV seropositivity')
CINAHL	("family planning" OR "birth control" OR contraceptive OR contraception OR fertility OR "reproductive counselling" OR "reproductive counseling" OR "reproductive planning" OR "birth spacing") AND (HIV-infected OR HIV-positive OR "women living with HIV" OR "people living with HIV" OR "HIV seropositive" OR "infected with HIV" OR "living with AIDS" OR "HIV seropositivity")
Sociological Abstracts	("family planning" OR "birth control" OR contraceptive OR contraception OR fertility OR "reproductive counselling" OR "reproductive counseling" OR "reproductive planning" OR "birth spacing") AND (HIV-infected OR HIV-positive OR "women living with HIV" OR "people living with HIV" OR "HIV seropositive" OR "infected with HIV" OR "living with AIDS" OR "HIV seropositivity")

## References

[1] De Cock KM, Fowler MG, Mercier E, et al. Prevention of mother-to-child HIV transmission in resource-poor countries: translating research into policy and practice. JAMA. 2000;283(9):1175–82. 10703780 10.1001/jama.283.9.1175

[2] Newell M-L. Prevention of mother-to-child transmission of HIV: challenges for the current decade. Bull World Health Organ. 2001;79(12):1138–44. 11799446 PMC2566720

[3] Thorne C, Newell M-L. Prevention of mother-to-child transmission of HIV infection. Curr Opin Infect Dis. 2004;17(3):247–52. 15166829 10.1097/00001432-200406000-00013

[4] Kourtis AP, Lee FK, Abrams EJ, Jamieson DJ, Bulterys M. Mother-to-child transmission of HIV-1: timing and implications for prevention. Lancet Infect Dis. 2006;6(11):726–32. 17067921 10.1016/S1473-3099(06)70629-6

[5] World Health Organization. *Strategic approaches to the prevention of HIV infection in infants: report of a WHO meeting, Morges, Switzerland, 20–22 March 2002.* Geneva, Switzerland: WHO; 2003. http://apps.who.int/iris/handle/10665/42723

[6] World Health Organization. *Guideline on when to start antiretroviral therapy and on pre-exposure prophylaxis for HIV.* Geneva, Switzerland: WHO; 2015. https://www.who.int/publications/i/item/9789241509565 26598776

[7] O’Reilly KR, Kennedy CE, Fonner VA, Sweat MD. Family planning counseling for women living with HIV: a systematic review of the evidence of effectiveness on contraceptive uptake and pregnancy incidence, 1990 to 2011. BMC Public Health. 2013;13:935. 24099177 10.1186/1471-2458-13-935 PMC3852503

[8] Page MJ, McKenzie JE, Bossuyt PM, et al. The PRISMA 2020 statement: an updated guideline for reporting systematic reviews. BMJ. 2021;372: n71. 33782057 10.1136/bmj.n71 PMC8005924

[9] World Bank. World Bank Country Classification: Country and Lending Groups. 2023. https://datahelpdesk.worldbank.org/knowledgebase/articles/906519. Accessed 1 Jan 2023.

[10] Kennedy CE, Fonner VA, Armstrong KA, et al. The Evidence Project risk of bias tool: assessing study rigor for both randomized and non-randomized intervention studies. Syst Rev. 2019;8(1):3. 30606262 10.1186/s13643-018-0925-0 PMC6317181

[11] Aradom HS, Sendo EG, Teshome GS, Dinagde NG, Demie TG. Factors associated with modern family planning use among women living with HIV who attended care and treatment clinics in Jigjiga town, Eastern Ethiopia. Ther Adv Reprod Health. 2020;14:2633494120976961. 33403360 10.1177/2633494120976961 PMC7739204

[12] Atukunda EC, Mugyenyi GR, Musiimenta A, et al. Structured and sustained family planning support facilitates effective use of postpartum contraception amongst women living with HIV in South Western Uganda: a randomized controlled trial. J Glob Health. 2021;11:04034. 34131487 10.7189/jogh.11.04034 PMC8183159

[13] Baumgartner JN, Green M, Weaver MA, et al. Integrating family planning services into HIV care and treatment clinics in Tanzania: evaluation of a facilitated referral model. Health Policy Plan. 2014;29(5):570–9. 23894070 10.1093/heapol/czt043 PMC4184336

[14] Dev R, Kohler P, Begnel E, et al. Contraceptive counseling experiences among women attending HIV care and treatment centers: a national survey in Kenya. Contraception. 2021;104(2):139–46. 33894251 10.1016/j.contraception.2021.04.011 PMC8286320

[15] Grossman D, Onono M, Newmann SJ, et al. Integration of family planning services into HIV care and treatment in Kenya: a cluster-randomized trial. AIDS. 2013;27(Suppl 1):S77-85. 24088687 10.1097/QAD.0000000000000035

[16] Hawkins L, Gertz AM, Badubi O, et al. Integration of family planning services into health care for HIV-positive women in Botswana. Int J Gynaecol Obst. 2021;152(2):208–14. 33145775 10.1002/ijgo.13464 PMC8084204

[17] Hoke T, Harries J, Crede S, et al. Expanding contraceptive options for PMTCT clients: a mixed methods implementation study in Cape Town, South Africa. Reprod Health. 2014;11(1):3–3. 24410922 10.1186/1742-4755-11-3 PMC3895666

[18] Joshi B, Velhal G, Chauhan S, Kulkarni R, Begum S. Linking HIV & family planning services to improve dual methods of contraception among women infected with HIV in Mumbai, Maharashtra, India. Indian J Med Res. 2016;143(4):464–73. 27377503 10.4103/0971-5916.184286 PMC4928553

[19] Kosgei RJ, Lubano KM, Shen C, et al. Impact of integrated family planning and HIV care services on contraceptive use and pregnancy outcomes: a retrospective cohort study. J Acquir Immune Defic Syndr. 2011;58(5):e121-126. 21963940 10.1097/QAI.0b013e318237ca80 PMC3779789

[20] Kuete M, Yuan HF, Tchoua Kemayou AL, et al. Scale up use of family planning services to prevent maternal transmission of HIV among discordant couples: a cross-sectional study within a resource-limited setting. Patient Pref Adherence. 2016;10:1967–77. 10.2147/PPA.S105624 PMC5055043 27757019

[21] Landolt NK, Achalapong J, Kosalaraksa P, et al. Strategies to improve the uptake of effective contraception in perinatally HIV-infected adolescents. J Virus Erad. 2017;3(3):152–6. 28758023 10.1016/S2055-6640(20)30334-4 PMC5518244

[22] Mantell JE, Cooper D, Exner TM, et al. Emtonjeni-A structural intervention to integrate sexual and reproductive health into public sector HIV Care in Cape Town, South Africa: results of a phase II study. AIDS Behav. 2017;21(3):905–22. 27807792 10.1007/s10461-016-1562-z PMC5552040

[23] McCarraher DR, Vance G, Gwarzo U, Taylor D, Chabikuli ON. Changes in contraceptive use following integration of family planning into ART Services in Cross River State, Nigeria. Stud Fam Plann. 2011;42(4):283–90. 22292247 10.1111/j.1728-4465.2011.00291.x

[24] Mudiope P, Musingye E, Makumbi CO, et al. Greater involvement of HIV-infected peer-mothers in provision of reproductive health services as “family planning champions” increases referrals and uptake of family planning among HIV-infected mothers. BMC Health Serv Res. 2017;17(1):444–444. 28655314 10.1186/s12913-017-2386-x PMC5488413

[25] Nabirye J, Matovu JKB, Bwanika JB, Makumbi F, Wanyenze RK. Missed opportunities for family planning counselling among HIV-positive women receiving HIV Care in Uganda. BMC Womens Health. 2020;20(1):91. 32370797 10.1186/s12905-020-00942-6 PMC7201557

[26] Onono M, Guzé MA, Grossman D, et al. Integrating family planning and HIV services in western Kenya: the impact on HIV-infected patients’ knowledge of family planning and male attitudes toward family planning. AIDS Care. 2015;27(6):743–52. 25634244 10.1080/09540121.2014.999744 PMC4366270

[27] Sarnquist CC, Moyo P, Stranix-Chibanda L, Chipato T, Kang JL, Maldonado YA. Integrating family planning and prevention of mother to child HIV transmission in Zimbabwe. Contraception. 2014;89(3):209–14. 24332254 10.1016/j.contraception.2013.11.003 PMC3965605

[28] Siveregi A, Dudley L, Makumucha C, Dlamini P, Moyo S, Bhembe S. Does counselling improve uptake of long-term and permanent contraceptive methods in a high HIV-prevalence setting? Afr J Prim Health Care Fam Med. 2015;7(1):779–779. 26842525 10.4102/phcfm.v7i1.779 PMC4685656

[29] Thyda L, Sineng S, Delvaux T, et al. Integration of family planning services in a peer-managed HIV care clinic serving most-at-risk populations in Phnom Penh, Cambodia. J Acquir Immune Defic Syndr. 2015;69(4):e120–6. 25850605 10.1097/QAI.0000000000000635

[30] Tusubira AK, Sebina Kibira SP, Makumbi FE. Modern contraceptive use among postpartum women living with HIV attending mother baby care points in Kabarole District, Uganda. MIDIRS Midwif Digest. 2020;30(3):402–402. 10.1186/s12905-020-00944-4 PMC7178756 32321480

[31] Tweya H, Feldacker C, Gugsa S, Phiri S. Contraceptive use and pregnancy rates among women receiving antiretroviral therapy in Malawi: a retrospective cohort study. Reprod Health. 2018. 10.1186/s12978-017-0440-0. 29426333 10.1186/s12978-017-0440-0 PMC5807743

[32] Vu L, Burnett-Zieman B, Banura C, et al. Increasing uptake of HIV, sexually transmitted infection, and family planning services, and reducing HIV-related risk behaviors among youth living with HIV in Uganda. J Adolesc Health. 2017;60(2s2):S22-s28. 28109336 10.1016/j.jadohealth.2016.09.007

[33] Wanyenze RK, Matovu JK, Kamya MR, Tumwesigye NM, Nannyonga M, Wagner GJ. Fertility desires and unmet need for family planning among HIV infected individuals in two HIV clinics with differing models of family planning service delivery. BMC Womens Health. 2015;15:5–5. 25627072 10.1186/s12905-014-0158-x PMC4320597

[34] Nkhoma L, Sitali DC, Zulu JM. Integration of family planning into HIV services: a systematic review. Ann Med. 2022;54(1):393–403. 35098814 10.1080/07853890.2021.2020893 PMC8812772

[35] Grant-Maidment T, Kranzer K, Ferrand RA. The effect of integration of family planning into HIV services on contraceptive use among women accessing HIV services in low and middle-income countries: a systematic review. Front Glob Women’s Health. 2022;3: 837358. 35284908 10.3389/fgwh.2022.837358 PMC8907733

[36] Pillai VK, Kelley AC. Men and family planning: toward a policy of male involvement. Polish Popul Rev. 1994;5:293–304. 12290097

[37] Anbesu EW, Aychiluhm SB, Kahsay ZH. Male involvement in family planning use and its determinants in Ethiopia: a systematic review and meta-analysis protocol. Syst Rev. 2022;11(1):19. 35105382 10.1186/s13643-022-01891-x PMC8805394

[38] Verbiest S, Malin CK, Drummonds M, Kotelchuck M. Catalyzing a reproductive health and social justice movement. Matern Child Health J. 2016;20(4):741–8. 26740226 10.1007/s10995-015-1917-5 PMC4792350

